# Is Quorum Sensing Interference a Viable Alternative to Treat *Pseudomonas aeruginosa* Infections?

**DOI:** 10.3389/fmicb.2016.01454

**Published:** 2016-09-14

**Authors:** Rodolfo García-Contreras

**Affiliations:** Department of Microbiology and Parasitology, Faculty of Medicine, National Autonomous University of MexicoMexico City, Mexico

**Keywords:** *Pseudomonas* infections, quorum sensing (QS), quorum quenching, resistance, clinical isolates

## Abstract

Quorum sensing (QS) coordinates the expression of multiple virulence factors in *Pseudomonas aeruginosa*; hence its inhibition has been postulated as a new alternative to treat its infections. In particular, QS interference approaches claim that they attenuate bacterial virulence without directly decreasing bacterial growth and suggest that *in vivo* the immune system would control the infections. Moreover, since *in vitro* experiments performed in rich medium demonstrate that interfering with QS decreases the production of virulence factors without affecting bacterial growth it was assumed than *in vivo* therapies will minimize the selection of resistant strains. Therefore, the underlying assumptions toward an effective implementation of a successful Quorum sensing interference (QSI) therapy for treating *P. aeruginosa* infections are that (i) QS only exerts important effects in the regulation of virulence genes but it does not affect metabolic processes linked to growth, (ii) the expression of virulence factors is only positively regulated by QS, (iii) inhibition of virulence factors *in vivo* do not affect bacterial growth, (iv) the immune system of the infected patients will be able to get rid of the infections, and (v) the therapy will be effective in the strains that are actively producing the infections. Nevertheless, for QSI in *P. aeruginosa*, substantial experimental evidence against the validity of most of these assumptions has accumulated during the past years, suggesting that a far better understanding of its virulence and its behavior during infections is needed in order to design truly solid QSI therapeutic alternatives to combat this remarkable pathogen.

## Introduction

*Pseudomonas aeruginosa* is a remarkable opportunistic pathogen that infects patients that are immunocompromised, have severe burns, cancer, or AIDS, are intubated and with prosthetic devices, and also those suffering from chronic affections like cystic fibrosis (CF). This bacterium is a major health problem worldwide being responsible of 10% of nosocomial infections (Antunes et al., 2010; Castillo-Juárez et al., 2015), since it is intrinsically resistant to several antimicrobials (Poole, 2011) and able to develop resistance against new ones, has a high biofilm production and produces an arsenal of virulence factors. One of the main mechanisms that controls the production of its virulence factors is quorum sensing (QS) which coordinates the expression of such factors once bacteria have reached a high population density, thus maximizing their chances to overcome the host defenses and establish the infection.

QS systems are common in bacterial pathogens; among Gram negatives, in addition to *P. aeruginosa, Acinetobacter baumannii* (Bhargava et al., 2010), *Escherichia coli* (Sperandio et al., 2002) Salmonella strains (Choi et al., 2007), and Vibrio strains (Zhu et al., 2002; Yang and Defoirdt, 2015) use them to coordinate their virulence. Hence, QS interference (QSI) or quorum quenching (QQ) is a strategy proposed to inhibit virulence as an alternative to treat the infections of several important bacterial pathogens (Castillo-Juárez et al., 2015).

In this opinion piece I focus in *P. aeruginosa*, one of the most studied organisms regarding QS and QSI, nevertheless what is exposed here may be also applicable to other bacterial pathogens.

*P. aeruginosa* is one of the more complex known bacterial pathogens; it possesses an ample genome and a high percentage of its genes are devoted to gene regulation (Stover et al., 2000). Regarding QS systems, it has a hierarchical architecture governed by the LasRI module which produces and senses N-3-oxo-dodecanoyl-L-Homoserine lactone and activates the expression of multiple virulence genes such as those producing elastase A and B, pyocyanin, alkaline and protease, and activating a second homoserine lactone (HSL) QS module known as RhlRI, which produces and senses N-butyryl-L-Homoserine lactone. RhlR bound to its autoinducer also activates directly some virulence genes like those encoding rhamnolipids and pyocyanin (Smith and Iglewski, 2003; Jimenez et al., 2012). In addition, *P. aeruginosa* also has a quinolone dependent system known as PQS, which is positively regulated by LasR and negatively by RhlR, forming a complex and intricate network (Jimenez et al., 2012; Lee and Zhang, 2015). In addition to the QS intrinsic components, several regulators such as GacA/GacS, QscR, Vfr, RpoN, and RpoS, influence the expression of QS dependent factors (Lee and Zhang, 2015).

The complex relationship between QS and virulence in *P. aeruginosa* had been recently evidenced by the fact that *lasR* deficient mutants (including clinical strains) growing at slow rates or in the stationary phase, overproduce pyocyanin due a lack of repression of the phenazine genes by RsaL, a negative transcriptional regulator positively controlled by LasR (Cabeen, 2014). Moreover, in *lasR* mutants, the activity of the *rhl* system is only delayed but not abolished, allowing significant production of pyocyanin, rhamnolipids and N-butyryl-L-Homoserine lactone (which are RhlR dependent) and even the production of the LasR dependent QS signals N-3-oxo-dodecanoyl-L-Homoserine lactone and PQS (Dekimpe and Deziel, 2009). These findings suggest that the inhibition of a particular component of the QS network, even the pivotal factor LasR, may be counteracted by the activation of alternative components of the network. Indeed, some recent studies have identified novel QS inhibitors for which RhlR and not LasR as the relevant *in vivo* target (O'Loughlin et al., 2013; Welsh et al., 2015).

In addition, recently it was discovered that an environmental strain (148 isolated from dolphin gastric juice) produces significant amounts of the QS-controlled virulence factors rhamnolipids and pyocyanin. Critically, this strain is virulent to mice, even without having a *lasR* gene and without producing N-3-oxo-dodecanoyl-L-Homoserine lactone. Hence, in this strain, the control of QS dependent virulence factors must be exerted by other regulators (Grosso-Becerra et al., 2014).

QS Interference could be achieved by the attenuation of the QS communication systems via: (i) the disruption of the QS receptors, (ii) the degradation of the autoinducers signals or (iii) the inhibition of the signal synthesis. Accordingly, several QS inhibitors or quorum quenchers (QQ) suitable for *P. aeruginosa* have been developed, under the assumptions that (i) the expression of virulence factors is only positively regulated by QS, (ii) the QQ will only exert significant effects in the regulation of virulence genes but not in metabolic processes linked to growth, hence avoiding or at least decreasing the generation of selective pressure that leads to resistance, (iii) the immune system of the infected patients will be able to get rid of the infections, and (iv) the therapy will be effective against the strains that are actively producing the infections. Nevertheless, substantial evidence against the validity of those assumptions has accumulated over the years.

## Quorum sensing influences metabolic processes and bacterial growth

Although the role of QS in *P. aeruginosa* virulence has been extensively studied, whether it influences global metabolism and cell growth is less explored. One possible reason for this bias is that at the transcriptomic level, few significant changes in metabolicrelated genes are found when QS proficient and deficient strains are compared or when QS inhibitors are administrated to QS proficient strains (Hentzer et al., 2003; Schuster et al., 2003; Wagner et al., 2003). Nevertheless, recently Davenport and coworkers found that the metabolome of a *lasI rhlI* double mutant (unable to produce HSL QS autoinducers) and that of its wild-type progenitor have a notorious divergence once the wild-type strain produced the highest levels of autoinducers; remarkably, around one third of all the metabolites identified changed (Davenport et al., 2015). This phenomenon is understandable since the wild-type strain devotes many of its resources to the production of costly virulence factors such as exoproteases, phenazines, and exopolysacharides, while, in contrast, the QS mutants uses the same resources for cell division. In agreement, the QS deficient mutants achieve higher growth yields than QS wild-type strains (Diggle et al., 2007).

Although the possible implications of the metabolic divergence in QS mutants on bacterial physiology and QS interference therapies is still unexplored, Davenport and coworkers demonstrated that important metabolic changes in the membrane metabolism of the wild-type strain are driven by QS upon entering the stationary phase. These modifications include increased fatty acid saturation, chain length, and cyclopropanation, which in turn, promote the generation of robust cell membranes. In contrast, the membranes of QS mutants do not have these modifications and are therefore more susceptible to stress.

In addition to these metabolic changes, several works have demonstrated that in *P. aeruginosa* and other bacteria, QS enhances the stress response (Bjarnsholt et al., 2005; Bhargava et al., 2014; de Oca-Mejia et al., 2015; García-Contreras et al., 2015a); for example, by upregulating antioxidant enzymes such as catalase, superoxide dismutase (Hassett et al., 1999) and NADPH-generating enzymes (García-Contreras et al., 2015a). This is important since during an infection, the immune system attacks bacteria by releasing reactive oxygen species, and at least *in vitro* oxidative stress is able to select functional QS systems as well as QS interference resistant mutants (García-Contreras et al., 2015a).

Moreover, growth of *P. aeruginosa* can be strongly dependent on QS either if adenosine or protein is used as sole carbon sources, since *nuh*, the gene encoding the nucleoside hydrolase, is under tight LasR control (Heurlier et al., 2005) and since the expression of exoproteases is under QS control (Diggle et al., 2007). During infections, adenosine utilization (Patel et al., 2007; Sheng et al., 2012) as well as the degradation of host proteins (Wretlind and Pavlovskis, 1983; Laarman et al., 2013) are important for bacterial virulence and survival.

All the above summarized facts demonstrate that QS is an important regulator of bacterial metabolism and physiology and suggest that QS interference will have the direct effect of decreasing bacterial growth and viability *in vivo*. In agreement, several *in vivo* infection studies have shown that when a QS interference therapy is able to increase animal survival (and decrease damage to the host), there is a significant decrease in bacterial counts in the infection sites (Wu et al., 2004; Christensen et al., 2007; Defoirdt et al., 2010; Jakobsen et al., 2011). The decrease in bacterial viability due QS interference may be an important potential source of *in vivo* selective pressure for the selection of bacterial resistance (García-Contreras et al., 2016).

## Potential increase in virulence upon quorum sensing interference

Although the production of multiple virulence factors in *P. aeruginosa* is positively regulated by QS, the virulence phenotype displayed by this organism is a complex combinatorial phenomenon that cannot be easily predicted with the identification of the presence or absence of a set of specific genes (Lee et al., 2006; Grosso-Becerra et al., 2014). In agreement, several studies had shown that clinical strains display a wide variety levels of virulence (Fenner et al., 2006; Lee et al., 2006; Garcia-Contreras et al., 2015b) and that environmental strains often conserve high virulence (Grosso-Becerra et al., 2014).

An overlooked fact about the role of QS in *P. aeruginosa* virulence was discovered in 2005 by Bleves and coworkers, who that found that in contrast to several other virulence traits, the expression of the type III secretion system (TTSS) in *P. aeruginosa* is negatively regulated by QS, specifically by RhlR and PqsR (Bleves et al., 2005; Kong et al., 2009). An important factor in such negative regulation of virulence genes by QS is that it requires low calcium levels, and hence several transcriptomics studies done in culture medium such as LB with relative high calcium levels fail to show this relationship. Since the TTSS is an important determinant of virulence in several animal infection models including pneumonia, peritonitis, bacteremia, burn infections, and keratitis (Hauser, 2009), the possibility that QS interfering therapies may be activating this system and therefore promoting virulence should not be ignored. Moreover, *P. aeruginosa* has five of the six known types of secretion systems present in Gram negative bacteria (all but type IV) and some of these systems, like type VI, are present in several copies in its genome (Bleves et al., 2010). The QS influence on the expression of these secretion systems is as of yet unknown, with the exceptions of the TTSS and the second type VI secretion system that are negatively regulated by QS (Sana et al., 2012).

More striking is the fact that QS interference using azithromycin selects the wild-type virulent phenotype against the less virulent *lasR* mutants in intubated patients colonized by *P. aeruginosa* (Kohler et al., 2010). This surprising fact is understandable since *lasR* mutants often appear and are selected in infections since they act as phenotypic cheaters that utilize the public goods such as exoproteases and siderophores produced by the cooperative wild-type individuals (Diggle et al., 2007; Sandoz et al., 2007). Hence, inhibiting QS removes the advantage of the *lasR* mutants and thereby selects the wild-type, thus a detrimental effect of QS interference in the long run could be to increase the prevalence of virulent genotypes in the nosocomial environment (Kohler et al., 2010).

Another key aspect of current QS inhibitors like furanones is that, depending on their concentration, they can activate rather than inhibit QS (Martinelli et al., 2004). Furthermore, related QS inhibitors such as synthetic HSLs can activate rather than inhibit some virulence factors (Welsh et al., 2015).

## Limitations of the current animal infection models for the study of quorum sensing interference

To date, several animal infection models have demonstrated that QS deficient mutants are much less virulent than their QS proficient parental strains; accordingly, QS interference promotes an increase in host survival and a decrease in damage and bacterial counts (Castillo-Juárez et al., 2015). The infection models used include arthropods like *Galleria mellonela*, nematodes such as *Caenorhabditis elegans*, fruit fly, and zebrafish which are valuable and informative but that do not accurately reflect human physiology. Moreover, when mice are used as a model, immune competent individuals are evaluated; hence, the fact that *P. aeruginosa* is a strict opportunistic pathogen that does not attack individuals with competent immune systems is overlooked, and so it is not clear if the immune systems of immunosuppressed patients will be able to clear the bacteria from infections upon QS interference treatments.

In addition to attacking immunosupressed individuals, *P. aeruginosa* is the major cause of death of CF patients; accordingly, there are several mice models that incorporate different mutations in the CF transmembrane conductance regulator (CTFR) protein and that are able to mimic some of the characteristics of the disease in humans. However, CF is a very complex disease and there are more than one thousand reported mutations in the *cftr* gene associated with the disease; hence, developing a murine model that closely resembles the human disease is challenging (Guilbault et al., 2007).

Most of the current models are suitable to study acute infections and only a few like the one developed in 2005 by Hoffmann and coworkers are optimized to simulate chronic CF infections (Hoffmann et al., 2005). Importantly, this model has been used to demonstrate that QS interference with azithromycin inhibits alginate production of the mucoid NH57388A strain *in vivo*, attenuating the damage produced to the host, but unfortunately it was not able to significantly increase mouse survival (Hoffmann et al., 2007).

Clinical studies have demonstrated that AZM treatment improves lung function in CF patients (Saiman et al., 2003); however, besides the QSI effect, azithromycin has bactericidal and anti-inflammatory effects; hence, the improvement of both mice and patients is likely due a combination of these effects rather than an exclusive consequence of the QSI properties of azithromycin. Therefore, testing of the effect of more specific QS interference molecules in these kind of models is needed in order to elucidate the potential of these therapies for CF patients.

Another kind of mouse model that more closely resembles the situation observed in humans is the thermally-induced injury model, which consists of producing a burn of second or third degree on the dorsal side of the mouse using hot water and subsequent inoculation of the burn. The utilization of this model has confirmed that QS-deficient mutants such as *lasR, lasI, rhlI, lasI rhlI*, and *pqsA* (Rumbaugh et al., 1999a,b; Lesic et al., 2007), have less virulence than the parental strains. Moreover, it has been used to test the effect of the halogenated anthranilic acid analogs 6FABA, 6CABA, and 4CABA which are inhibitors of the synthesis of quinolone QS signals. These compounds, at the doses administrated, decrease mouse mortality significantly. Nevertheless, they were tested at a single dose (Lesic et al., 2007), so it is unknown if they exert dose response effects. This is not trivial since as mentioned before, QS inhibitors may act as QS activators depending on the used doses (Martinelli et al., 2004).

## Insensibility of some clinical strains to current quorum quenchers

Before 2010 it was assumed than QS interference will be impervious or at least less susceptible to promote bacterial resistance than conventional antibiotic therapies (Defoirdt et al., 2010). In 2011, it was demonstrated that *P. aeruginosa* acquires resistance easily against the canonical HSL-dependant quorum quencher furanone C-30, by activating the multidrug efflux pump MexAB-OprM (Maeda et al., 2012). The activation of this pump is mediated by mutations disrupting the transcriptional repressors MexR and NalC, and such mutations are common in clinical isolates, presumably since they are selected by intense antibiotic treatments (Tomas et al., 2010). As expected, these clinical isolates are resistant against C-30 (Maeda et al., 2012).

Recent studies have demonstrated that C-30 resistance is common in multidrug resistant strains. Importantly, resistance against other QQ compounds like 5-fluorouracil is also present in some clinical isolates and some antibiotic sensitive strains are also resistant to C-30 by a decrease in the compound uptake. More strikingly, some of these strains produce higher amounts of the virulence factors in the presence of the furanone than in basal conditions (García-Contreras et al., 2013a,b; Garcia-Contreras et al., 2015b).

This suggests that if eventually QSI therapies are implemented in the clinic, they likely would not be effective against all the strains present in infections (García-Contreras et al., 2013a; Kalia et al., 2013), and that crucially they may fail to inhibit those strains with active antibiotic efflux pumps. Indeed, it was recently proposed that bacteria resistant to several QS inhibitors may be selected by QSI treatments (Koul et al., 2016).

## Perspectives

Since recent evidence has demonstrated that QS is linked to basal metabolism and growth, it will be important to evaluate QS interference and its effect in bacteria growing in medium that more closely simulates the composition of the environment during infection (Palmer et al., 2007; Garcia-Contreras et al., 2015b), with attention on key metabolites that influence virulence and QS such as iron (Sokol and Woods, 1984; Mittal et al., 2008; Hazan et al., 2010), calcium (Sarkisova et al., 2005), phosphate (Zaborin et al., 2009), and adenosine (Patel et al., 2007; Sheng et al., 2012). Further, metabolomic and proteomic studies may shed light about the role of QS in bacterial physiology under *in vivo*-like conditions. In addition, the utilization of new technologies like microfluidics such as cell culture chips (organ-on-a-chip) would enable the interrogation of the roles of signaling, social cheating, mass transfer, and spatial organization in well-defined geometries, which is virtually impossible to asses at this moment using animal models.

Importantly, additional research about the ways *P. aeruginosa* may achieve resistance against QQ is needed in order to generate effective combination therapies less prone to the selection of resistance. One possibility is to exploit the fact that QS interference renders *P. aeruginosa* more sensitive to stress, including antibiotics and the effects of the immune system; hence, it is attractive to test if the combination of QS interference, antibiotic therapies and immunotherapy to improve the outcome of the therapies.

Another area of research that should be encouraged is the role of QS independent virulence factors and those negatively regulated by QS such as TTSS in infections and the effect of QS interference on these virulence determinants. Also, study of QS networks and the-virulence of environmental and clinical strains is needed since current evidence indicates these are highly variable (Grosso-Becerra et al., 2014; Garcia-Contreras et al., 2015b) and that a specific QQ compound will not always be effective against all clinical strains. Also, more effort should be devoted to the study of the implementation of these therapies, since most of them are able to attenuate infections when the QQ compound is administrated shortly before the inoculation (having possible prophylactic effects), but the value of such therapies as a possible cure for an established infection should be better addressed.

Taking the available evidence together, it seems QS interference may be a valuable tool to combat *P. aeruginosa* infections; however, using QS inhibition as the sole therapy may not be an efficient strategy due several potential drawbacks. Hence, before QSI applications are used in the clinic, it is advisable to improve our current understanding of *P. aeruginosa* virulence with an emphasis on clinical strains and also on further defining the roles of QS in the physiology of this remarkable complex pathogen.

## Author contributions

The author confirms being the sole contributor of this work and approved it for publication.

## Funding

This work was supported by grants from SEP/CONACyT-México No. 152794, CONACYT Problemas Nacionales 2015 No. 2015-01-402 and PAPIIT-UNAM No. IA201116.

### Conflict of interest statement

The author declares that the research was conducted in the absence of any commercial or financial relationships that could be construed as a potential conflict of interest.

## References

[B1] Antunes L. C. Ferreira R. B. Buckner M. M. Finlay B. B. (2010). Quorum sensing in bacterial virulence. Microbiology 156, 2271–2282. 10.1099/mic.0.038794-0 20488878

[B2] Bhargava N. Sharma P. Capalash N. (2010). Quorum sensing in Acinetobacter: an emerging pathogen. Crit. Rev. Microbiol. 36, 349–360. 10.3109/1040841X.2010.512269 20846031

[B3] Bhargava N. Sharma P. Capalash N. (2014). Pyocyanin stimulates quorum sensing-mediated tolerance to oxidative stress and increases persister cell populations in *Acinetobacter baumannii*. Infect. Immun. 82, 3417–3425. 10.1128/IAI.01600-14 24891106 PMC4136213

[B4] Bjarnsholt T. Jensen P. Ø. Burmølle M. Hentzer M. Haagensen J. A. Hougen H. P.. (2005). *Pseudomonas aeruginosa* tolerance to tobramycin, hydrogen peroxide and polymorphonuclear leukocytes is quorum-sensing dependent. Microbiology 151, 373–383. 10.1099/mic.0.27463-0 15699188

[B5] Bleves S. Soscia C. Nogueira-Orlandi P. Lazdunski A. Filloux A. (2005). Quorum sensing negatively controls type III secretion regulon expression in *Pseudomonas aeruginosa* PAO1. J. Bacteriol. 187, 3898–3902. 10.1128/JB.187.11.3898-3902.2005 15901720 PMC1112058

[B6] Bleves S. Viarre V. Salacha R. Michel G. P. Filloux A. Voulhoux R. (2010). Protein secretion systems in *Pseudomonas aeruginosa*: a wealth of pathogenic weapons. Int. J. Med. Microbiol. 300, 534–543. 10.1016/j.ijmm.2010.08.005 20947426

[B7] Cabeen M. T. (2014). Stationary phase-specific virulence factor overproduction by a *lasR* mutant of *Pseudomonas aeruginosa*. PLoS ONE 9:e88743. 10.1371/journal.pone.0088743 24533146 PMC3923063

[B8] Castillo-Juárez I. Maeda T. Mandujano-Tinoco E. A. Tomas M. Párez-Eretza B. Garcia-Contreras S. J.. (2015). Role of quorum sensing in bacterial infections. World J. Clin. Cases 3, 575–598. 10.12998/wjcc.v3.i7.575 26244150 PMC4517333

[B9] Choi J. Shin D. Ryu S. (2007). Implication of quorum sensing in *Salmonella enterica* serovar typhimurium virulence: the luxS gene is necessary for expression of genes in pathogenicity island 1. Infect. Immun. 75, 4885–4890. 10.1128/IAI.01942-06 17620352 PMC2044537

[B10] Christensen L. D. Moser C. Jensen P. Ø. Rasmussen T. B. Christophersen L. Kjelleberg S.. (2007). Impact of *Pseudomonas aeruginosa* quorum sensing on biofilm persistence in an *in vivo* intraperitoneal foreign-body infection model. Microbiology 153, 2312–2320. 10.1099/mic.0.2007/006122-0 17600075

[B11] Davenport P. W. Griffin J. L. Welch M. (2015). Quorum sensing is accompanied by global metabolic changes in the opportunistic human pathogen *Pseudomonas aeruginosa*. J. Bacteriol. 197, 2072–2082. 10.1128/JB.02557-14 25868647 PMC4438216

[B12] Defoirdt T. Boon N. Bossier P. (2010). Can bacteria evolve resistance to quorum sensing disruption? PLoS Pathog. 6:e1000989. 10.1371/journal.ppat.1000989 20628566 PMC2900297

[B13] Dekimpe V. Deziel E. (2009). Revisiting the quorum-sensing hierarchy in *Pseudomonas aeruginosa*: the transcriptional regulator RhlR regulates LasR-specific factors. Microbiology 155, 712–723. 10.1099/mic.0.022764-0 19246742

[B14] de Oca-Mejia M. M. Castillo-Juarez I. Martínez-Vázquez M. Soto-Hernandez M. García-Contreras R. (2015). Influence of quorum sensing in multiple phenotypes of the bacterial pathogen *Chromobacterium violaceum*. Pathog. Dis. 73, 1–4. 10.1093/femspd/ftu019 25722489

[B15] Diggle S. P. Griffin A. S. Campbell G. S. West S. A. (2007). Cooperation and conflict in quorum-sensing bacterial populations. Nature 450, 411–414. 10.1038/nature06279 18004383

[B16] Fenner L. Richet H. Raoult D. Papazian L. Martin C. La Scola B. (2006). Are clinical isolates of *Pseudomonas aeruginosa* more virulent than hospital environmental isolates in amebal co-culture test? Crit. Care Med. 34, 823–828. 10.1097/01.CCM.0000201878.51343.F1 16505663

[B17] García-Contreras R. Maeda T. Wood T. K. (2013a). Resistance to quorum-quenching compounds. Appl. Environ. Microbiol. 79, 6840–6846. 10.1128/AEM.02378-13 24014536 PMC3811534

[B18] García-Contreras R. Maeda T. Wood T. K. (2016). Can resistance against quorum-sensing interference be selected? ISME J. 10, 4–10. 10.1038/ismej.2015.84 26023871 PMC4681860

[B19] García-Contreras R. Martinez-Vazquez M. Velazquez Guadarrama N. Villegas Paneda A. G. Hashimoto T. Maeda T.. (2013b). Resistance to the quorum-quenching compounds brominated furanone C-30 and 5-fluorouracil in *Pseudomonas aeruginosa* clinical isolates. Pathog. Dis. 68, 8–11. 10.1111/2049-632X.12039 23620228

[B20] García-Contreras R. Nunez-López L. Jasso-Chávez R. Kwan B. W. Belmont J. A. Rangel-Vega A.. (2015a). Quorum sensing enhancement of the stress response promotes resistance to quorum quenching and prevents social cheating. ISME J. 9, 115–125. 10.1038/ismej.2014.98 24936763 PMC4274435

[B21] Garcia-Contreras R. Perez-Eretza B. Jasso-Chavez R. Lira-Silva E. Roldan-Sanchez J. A. Gonzalez-Valdez A.. (2015b). High variability in quorum quenching and growth inhibition by furanone C-30 in *Pseudomonas aeruginosa* clinical isolates from cystic fibrosis patients. Pathog. Dis. 73:ftv040. 10.1093/femspd/ftv040 26048733

[B22] Grosso-Becerra M. V. Santos-Medellin C. Gonzalez-Valdez A. Mendez J. L. Delgado G. Morales-Espinosa R.. (2014). *Pseudomonas aeruginosa* clinical and environmental isolates constitute a single population with high phenotypic diversity. BMC Genomics 15:318. 10.1186/1471-2164-15-318 24773920 PMC4234422

[B23] Guilbault C. Saeed Z. Downey G. P. Radzioch D. (2007). Cystic fibrosis mouse models. Am. J. Respir. Cell Mol. Biol. 36, 1–7. 10.1165/rcmb.2006-0184TR 16888286

[B24] Hassett D. J. Ma J. F. Elkins J. G. McDermott T. R. Ochsner U. A. West S. E.. (1999). Quorum sensing in *Pseudomonas aeruginosa* controls expression of catalase and superoxide dismutase genes and mediates biofilm susceptibility to hydrogen peroxide. Mol. Microbiol. 34, 1082–1093. 10.1046/j.1365-2958.1999.01672.x 10594832

[B25] Hauser A. R. (2009). The type III secretion system of *Pseudomonas aeruginosa*: infection by injection. Nat. Rev. Microbiol. 7, 654–665. 10.1038/nrmicro2199 19680249 PMC2766515

[B26] Hazan R. He J. Xiao G. Dekimpe V. Apidianakis Y. Lesic B.. (2010). Homeostatic interplay between bacterial cell-cell signaling and iron in virulence. PLoS Pathog. 6:e1000810. 10.1371/journal.ppat.1000810 20300606 PMC2837411

[B27] Hentzer M. Wu H. Andersen J. B. Riedel K. Rasmussen T. B. Bagge N.. (2003). Attenuation of *Pseudomonas aeruginosa* virulence by quorum sensing inhibitors. EMBO J. 22, 3803–3815. 10.1093/emboj/cdg366 12881415 PMC169039

[B28] Heurlier K. Dénervaud V. Haenni M. Guy L. Krishnapillai V. Haas D. (2005). Quorum-sensing-negative (*lasR*) mutants of *Pseudomonas aeruginosa* avoid cell lysis and death. J. Bacteriol. 187, 4875–4883. 10.1128/JB.187.14.4875-4883.2005 15995202 PMC1169536

[B29] Hoffmann N. Lee B. Hentzer M. Rasmussen T. B. Song Z. Johansen H. K.. (2007). Azithromycin blocks quorum sensing and alginate polymer formation and increases the sensitivity to serum and stationary-growth-phase killing of *Pseudomonas aeruginosa* and attenuates chronic *P. aeruginosa* lung infection in Cftr(-/-) mice. Antimicrob. Agents Chemother. 51, 3677–3687. 10.1128/AAC.01011-06 17620382 PMC2043275

[B30] Hoffmann N. Rasmussen T. B. Jensen P. Ø. Stub C. Hentzer M. Molin S.. (2005). Novel mouse model of chronic *Pseudomonas aeruginosa* lung infection mimicking cystic fibrosis. Infect. Immun. 73, 2504–2514. 10.1128/IAI.73.4.2504-2514.2005 15784597 PMC1087399

[B31] Jakobsen T. H. van Gennip M. Phipps R. K. Shanmugham M. S. Christensen L. D. Alhede M.. (2011). Ajoene, a sulfur-rich molecule from garlic, inhibits genes controlled by quorum sensing. Antimicrob. Agents Chemother. 56, 2314–2325. 10.1128/AAC.05919-11 22314537 PMC3346669

[B32] Jimenez P. N. Koch G. Thompson J. A. Xavier K. B. Cool R. H. Quax W. J. (2012). The multiple signaling systems regulating virulence in *Pseudomonas aeruginosa*. Microbiol. Mol. Biol. Rev. 76, 46–65. 10.1128/MMBR.05007-11 22390972 PMC3294424

[B33] Kalia V. C. Wood T. K. Kumar P. (2013). Evolution of resistance to quorum-sensing inhibitors. Microb. Ecol. 68, 13–23. 10.1007/s00248-013-0316-y 24194099 PMC4012018

[B34] Kohler T. Perron G. G. Buckling A. van Delden C. (2010). Quorum sensing inhibition selects for virulence and cooperation in *Pseudomonas aeruginosa*. PLoS Pathog. 6:e1000883. 10.1371/journal.ppat.1000883 20463812 PMC2865528

[B35] Kong W. Liang H. Shen L. Duan K. (2009). [Regulation of type III secretion system by Rhl and PQS quorum sensing systems in *Pseudomonas aeruginosa*]. Wei Sheng Wu Xue Bao 49, 1158–1164. Available online at: http://journals.im.ac.cn/actamicrocn/ch/reader/view_abstract.aspx?file_no=909-5 20030052

[B36] Koul S. Prakash J. Mishra A. Kalia V. C. (2016). Potential emergence of multi-quorum sensing inhibitor resistant (MQSIR) bacteria. Indian J. Microbiol. 56, 1–18. 10.1007/s12088-015-0558-0 26843692 PMC4729740

[B37] Laarman A. J. Bardoel B. W. Ruyken M. Fernie J. Milder F. J. van Strijp J. A.. (2013). *Pseudomonas aeruginosa* alkaline protease blocks complement activation via the classical and lectin pathways. J. Immunol. 188, 386–393. 10.4049/jimmunol.1102162 22131330

[B38] Lee D. G. Urbach J. M. Wu G. Liberati N. T. Feinbaum R. L. Miyata S.. (2006). Genomic analysis reveals that *Pseudomonas aeruginosa* virulence is combinatorial. Genome Biol. 7:R90. 10.1186/gb-2006-7-10-r90 17038190 PMC1794565

[B39] Lee J. Zhang L. (2015). The hierarchy quorum sensing network in *Pseudomonas aeruginosa*. Protein Cell 6, 26–41. 10.1007/s13238-014-0100-x 25249263 PMC4286720

[B40] Lesic B. Lépine F. Déziel E. Zhang J. Zhang Q. Padfield K.. (2007). Inhibitors of pathogen intercellular signals as selective anti-infective compounds. PLoS Pathog. 3, 1229–1239. 10.1371/journal.ppat.0030126 17941706 PMC2323289

[B41] Maeda T. García-Contreras R. Pu M. Sheng L. Garcia L. R. Tomás M.. (2012). Quorum quenching quandary: resistance to antivirulence compounds. ISME J. 6, 493–501. 10.1038/ismej.2011.122 21918575 PMC3280137

[B42] Martinelli D. Grossmann G. Sequin U. Brandl H. Bachofen R. (2004). Effects of natural and chemically synthesized furanones on quorum sensing in *Chromobacterium violaceum*. BMC Microbiol. 4:25. 10.1186/1471-2180-4-25 15233843 PMC509243

[B43] Mittal R. Sharma S. Chhibber S. Harjai K. (2008). Iron dictates the virulence of *Pseudomonas aeruginosa* in urinary tract infections. J. Biomed. Sci. 15, 731–741. 10.1007/s11373-008-9274-7 18688758

[B44] O'Loughlin C. T. Miller L. C. Siryaporn A. Drescher K. Semmelhack M. F. Bassler B. L. (2013). A quorum-sensing inhibitor blocks *Pseudomonas aeruginosa* virulence and biofilm formation. Proc. Natl. Acad. Sci. U.S.A. 110, 17981–17986. 10.1073/pnas.1316981110 24143808 PMC3816427

[B45] Palmer K. L. Aye L. M. Whiteley M. (2007). Nutritional cues control *Pseudomonas aeruginosa* multicellular behavior in cystic fibrosis sputum. J. Bacteriol. 189, 8079–8087. 10.1128/JB.01138-07 17873029 PMC2168676

[B46] Patel N. J. Zaborina O. Wu L. Wang Y. Wolfgeher D. J. Valuckaite V.. (2007). Recognition of intestinal epithelial HIF-1alpha activation by *Pseudomonas aeruginosa*. Am. J. Physiol. Gastrointest. Liver Physiol. 292, G134–G142. 10.1152/ajpgi.00276.2006 16901993 PMC2694754

[B47] Poole K. (2011). *Pseudomonas aeruginosa*: resistance to the max. Front. Microbiol. 2:65. 10.3389/fmicb.2011.00065 21747788 PMC3128976

[B48] Rumbaugh K. P. Griswold J. A. Hamood A. N. (1999a). Contribution of the regulatory gene *lasR* to the pathogenesis of infection of burned mice. J. Burn Care Rehabil. 20, 42–49. 10.1097/00004630-199901001-00008 9934636

[B49] Rumbaugh K. P. Griswold J. A. Iglewski B. H. Hamood A. N. (1999b). Contribution of quorum sensing to the virulence of *Pseudomonas aeruginosa* in burn wound infections. Infect. Immun. 67, 5854–5862. 10531240 10.1128/iai.67.11.5854-5862.1999 PMC96966

[B50] Saiman L. Marshall B. C. Mayer-Hamblett N. Burns J. L. Quittner A. L. Cibene D. A.. (2003). Azithromycin in patients with cystic fibrosis chronically infected with *Pseudomonas aeruginosa*: a randomized controlled trial. JAMA 290, 1749–1756. 10.1001/jama.290.13.1749 14519709

[B51] Sana T. G. Hachani A. Bucior I. Soscia C. Garvis S. Termine E.. (2012). The second type VI secretion system of *Pseudomonas aeruginosa* strain PAO1 is regulated by quorum sensing and Fur and modulates internalization in epithelial cells. J. Biol. Chem. 287, 27095–27105. 10.1074/jbc.M112.376368 22665491 PMC3411052

[B52] Sandoz K. M. Mitzimberg S. M. Schuster M. (2007). Social cheating in *Pseudomonas aeruginosa* quorum sensing. Proc. Natl. Acad. Sci. U.S.A. 104, 15876–15881. 10.1073/pnas.0705653104 17898171 PMC2000394

[B53] Sarkisova S. Patrauchan M. A. Berglund D. Nivens D. E. Franklin M. J. (2005). Calcium-induced virulence factors associated with the extracellular matrix of mucoid *Pseudomonas aeruginosa* biofilms. J. Bacteriol. 187, 4327–4337. 10.1128/JB.187.13.4327-4337.2005 15968041 PMC1151780

[B54] Schuster M. Lostroh C. P. Ogi T. Greenberg E. P. (2003). Identification, timing, and signal specificity of *Pseudomonas aeruginosa* quorum-controlled genes: a transcriptome analysis. J. Bacteriol. 185, 2066–2079. 10.1128/JB.185.7.2066-2079.2003 12644476 PMC151497

[B55] Sheng L. Pu M. Hegde M. Zhang Y. Jayaraman A. Wood T. K. (2012). Interkingdom adenosine signal reduces *Pseudomonas aeruginosa* pathogenicity. Microb. Biotechnol. 5, 560–572. 10.1111/j.1751-7915.2012.00338.x 22414222 PMC3815332

[B56] Smith R. S. Iglewski B. H. (2003). *Pseudomonas aeruginosa* quorum sensing as a potential antimicrobial target. J. Clin. Invest. 112, 1460–1465. 10.1172/JCI200320364 14617745 PMC259138

[B57] Sokol P. A. Woods D. E. (1984). Relationship of iron and extracellular virulence factors to *Pseudomonas aeruginosa* lung infections. J. Med. Microbiol. 18, 125–133. 10.1099/00222615-18-1-125 6431109

[B58] Sperandio V. Li C. C. Kaper J. B. (2002). Quorum-sensing *Escherichia coli* regulator A: a regulator of the LysR family involved in the regulation of the locus of enterocyte effacement pathogenicity island in enterohemorrhagic *E. coli*. Infect. Immun. 70, 3085–3093. 10.1128/IAI.70.6.3085-3093.2002 12011002 PMC127966

[B59] Stover C. K. Pham X. Q. Erwin A. L. Mizoguchi S. D. Warrener P. Hickey M. J.. (2000). Complete genome sequence of *Pseudomonas aeruginosa* PAO1, an opportunistic pathogen. Nature 406, 959–964. 10.1038/35023079 10984043

[B60] Tomás M. Doumith M. Warner M. Turton J. F. Beceiro A. Bou G.. (2010). Efflux pumps, OprD porin, AmpC beta-lactamase, and multiresistance in *Pseudomonas aeruginosa* isolates from cystic fibrosis patients. Antimicrob. Agents Chemother. 54, 2219–2224. 10.1128/AAC.00816-09 20194693 PMC2863613

[B61] Wagner V. E. Bushnell D. Passador L. Brooks A. I. Iglewski B. H. (2003). Microarray analysis of *Pseudomonas aeruginosa* quorum-sensing regulons: effects of growth phase and environment. J. Bacteriol. 185, 2080–2095. 10.1128/JB.185.7.2080-2095.2003 12644477 PMC151498

[B62] Welsh M. A. Eibergen N. R. Moore J. D. Blackwell H. E. (2015). Small molecule disruption of quorum sensing cross-regulation in *Pseudomonas aeruginosa* causes major and unexpected alterations to virulence phenotypes. J. Am. Chem. Soc. 137, 1510–1519. 10.1021/ja5110798 25574853 PMC4372995

[B63] Wretlind B. Pavlovskis O. R. (1983). *Pseudomonas aeruginosa* elastase and its role in pseudomonas infections. Rev. Infect. Dis. 5(Suppl. 5), S998–S1004. 10.1093/clinids/5.supplement_5.s998 6419322

[B64] Wu H. Song Z. Hentzer M. Andersen J. B. Molin S. Givskov M.. (2004). Synthetic furanones inhibit quorum-sensing and enhance bacterial clearance in *Pseudomonas aeruginosa* lung infection in mice. J. Antimicrob. Chemother. 53, 1054–1061. 10.1093/jac/dkh223 15117922

[B65] Yang Q. Defoirdt T. (2015). Quorum sensing positively regulates flagellar motility in pathogenic *Vibrio harveyi*. Environ. Microbiol. 17, 960–968. 10.1111/1462-2920.12420 24528485

[B66] Zaborin A. Romanowski K. Gerdes S. Holbrook C. Lepine F. Long J.. (2009). Red death in *Caenorhabditis elegans* caused by *Pseudomonas aeruginosa* PAO1. Proc. Natl. Acad. Sci. U.S.A. 106, 6327–6332. 10.1073/pnas.0813199106 19369215 PMC2669342

[B67] Zhu J. Miller M. B. Vance R. E. Dziejman M. Bassler B. L. Mekalanos J. J. (2002). Quorum-sensing regulators control virulence gene expression in *Vibrio cholerae*. Proc. Natl. Acad. Sci. U.S.A. 99, 3129–3134. 10.1073/pnas.052694299 11854465 PMC122484

